# 849. Histopathology of Cutaneous Invasive Fungal Infections in a Tertiary Cancer Center: Causes, Discordance with Culture, and Histopathologic Determinants of Outcome

**DOI:** 10.1093/ofid/ofad500.894

**Published:** 2023-11-27

**Authors:** Pavandeep Gill, Sebastian Wurster, Jeffrey Tarrand, Xinyang Jiang, Jing Ning, Ying Jiang, Phyu P Aung, Woo Cheal Cho, Jonathan L Curry, Carlos A Torres-Cabala, Doina Ivan, Victor G Prieto, Dimitrios P Kontoyiannis, Priyadharsini Nagarajan

**Affiliations:** University of British Columbia and Royal Jubilee Hospital, Victoria, British Columbia, Canada; The University of Texas MD Anderson Cancer Center, Houston, Texas; The University of Texas MD Anderson Cancer Center, Houston, Texas; The University of Texas MD Anderson Cancer Center, Houston, Texas; The University of Texas MD Anderson Cancer Center, Houston, Texas; The University of Texas MD Anderson Cancer Center, Houston, Texas; The University of Texas MD Anderson Cancer Center, Houston, Texas; The University of Texas MD Anderson Cancer Center, Houston, Texas; The University of Texas MD Anderson Cancer Center, Houston, Texas; The University of Texas MD Anderson Cancer Center, Houston, Texas; The University of Texas MD Anderson Cancer Center, Houston, Texas; The University of Texas MD Anderson Cancer Center, Houston, Texas; The University of Texas MD Anderson Cancer Center, Houston, Texas; The University of Texas MD Anderson Cancer Center, Houston, Texas

## Abstract

**Background:**

Cutaneous invasive fungal infections CIFIs (primary or secondary to hematogenous seeding) are frequent and often fatal in immunocompromised cancer patients (pts). There is a paucity of studies on the prognostic significance and concordance of histopathologic features with cultures.

**Methods:**

We reviewed all pts with histologically diagnosed CIFIs at MD Anderson Cancer Center (06/2016-06/2020). Demographic, clinical, histopathologic [organism, distribution (dermis/subcutis, blood vessels/nerves/epidermis), density of fungi and host response (inflammation, fibrosis)], culture, and outcome (all-cause mortality) data were recorded.

**Results:**

We identified 61 pts (median age: 60 years, range: 8-81); 37 (61%) were male. Most had hematologic malignancy (n=58, 95%), especially acute leukemia (n = 40, 66%). CIFI was primary in 53 pts (87%), with acute onset (≤ 1 week) in 66% of pts; 37 pts (61%) had multiple skin lesions. Fungal organisms were seen on H&E-stained sections in 47 cases (77%), whereas ancillary studies (GMS/PAS) were required in 14 cases (23%). Of the 59 concurrent microbiology cultures, only 43 (73%) were positive. In 16 cases, fungal order/genus was identified by both histopathology and culture; 13/16 (81%) were concordant (Fleiss’ kappa 0.67, Fig 1A). The causative fungal order/genus was determined in 55 pts (90%), most commonly *Fusarium* (n = 22, 36%) or Mucorales (n = 12, 20%, Fig. 1B). Angiotropism was most frequently associated with *Fusarium* (19/22, 88%), and neurotropism with Mucorales (8/12, 67%, Table 1). Eighty-four-day all-cause mortality rate was 62% (66% and 50% in CIFIs caused by molds and yeasts, respectively). Fungal angiotropism (p = 0.001, Fig 2A) and neurotropism (p < 0.001, Fig 2B) were associated with significantly increased mortality, while lymphocytic inflammation, seen only in 20%, was associated with reduced mortality (p = 0.024, Fig 2C).

**Figure 1**

**Figure:**
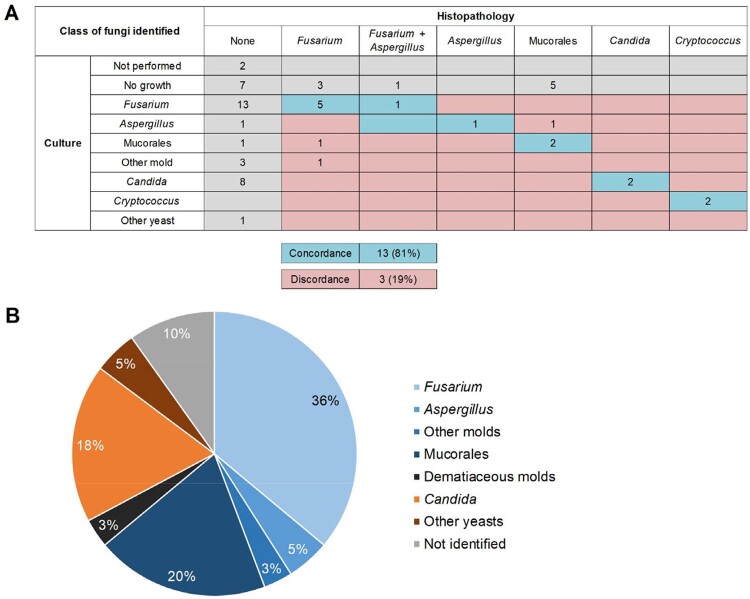

(A) Concordance of histopathologically determined and cultured fungal order/genus. Fleiss’ kappa 0.67 (“substantial agreement”), p < 0.001. (B) Distribution of causative fungal pathogens.

**Figure 2**

**Figure:**
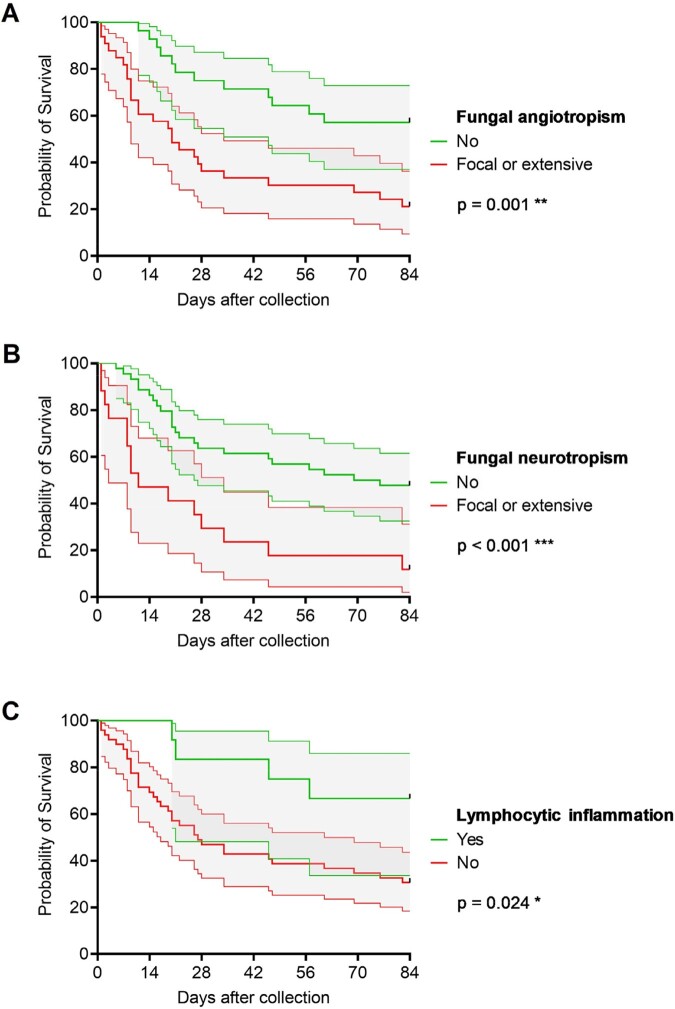

Histopathological features significantly associated with 84-day all-cause mortality in CIFI patients. Error bands denote 95% confidence interval. Mantel-Cox log-rank test.

**Table 1**

**Figure:**
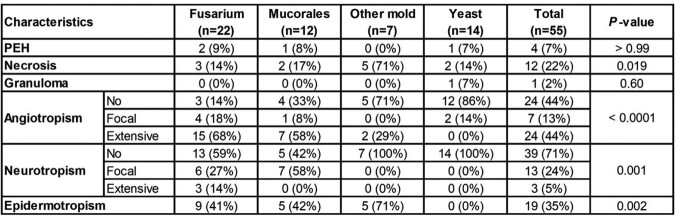

Association between type of organism and histopathological characteristics. Six patients with no identified organism were excluded. Fisher’s exact test. Abbreviation: PEH = pseudoepitheliomatous hyperplasia.

**Conclusion:**

CIFIs have poor prognosis, especially when caused by molds and if fungal angio-/neurotropism is identified. Inflammation may be associated with better prognosis. Since cultures might be false negative (27%) or discordant (19%), more efforts are needed for culture-independent molecular detection of fungi. Incorporation of histopathologic features might inform prognostic risk stratification.

**Disclosures:**

**Dimitrios P. Kontoyiannis, MD, MS, ScD, PhD**, AbbVie: Board Member|Astellas: Grant/Research Support|Cidara: Board Member|Gilead: Grant/Research Support|Merck: Advisor/Consultant|Scynexis/MSGERC: Board Member

